# Adjuvant immune checkpoint inhibitors across solid tumors: a meta-analysis of phase II-III randomized trials

**DOI:** 10.3389/fonc.2026.1903546

**Published:** 2026-09-07

**Authors:** Mariam Grazia Polito, Luisana Sisca, Davide Caruso, Antonella Cosimati, Carla Adriana Ramirez Reinaga, Etien Leka, Daniele Santini, Gian Paolo Spinelli

**Affiliations:** 1 UOC Oncologia Territoriale – ASL Latina – CDS Aprilia, La Sapienza Università Di Roma Polo Pontino, Latina, Italy; 2 Department of Medical Oncology, Fondazione Policlinico Universitario Campus Bio-Medico, Rome, Italy; 3 Department of Medical and Surgical Science and Biotechnology, Università “La Sapienza”, Rome, Italy

**Keywords:** adjuvant therapy, cancer immunotherapy, immune checkpoint inhibitor (ICI), meta-analysis, survival outcomes

## Abstract

**Introduction:**

Immunotherapy has reshaped the adjuvant landscape, with several immune checkpoint inhibitors (ICIs) approved in different tumor types. This meta-analysis evaluates the benefit of adjuvant ICIs across solid tumors.

**Methods:**

A systematic literature search of PubMed, Embase, the Cochrane Central Register of Controlled Trials (CENTRAL), and the proceedings of the American Society of Clinical Oncology (ASCO) and the European Society for Medical Oncology (ESMO) was performed from January 2015 to June 2026 in accordance with PRISMA to identify phase II and III randomized controlled trials (RCTs) testing ICIs versus non-ICI controls in the adjuvant setting. Trials with a single arm, local treatments, or combinations of ICIs with non-ICI systemic treatments (including chemotherapy, radiotherapy, targeted therapies, or vaccines) were excluded. Eligible comparisons included ICI-based adjuvant strategies versus randomized comparator arms defined by the original trial design. Trials comparing an ICI strategy with an alternative active immunotherapy regimen were considered eligible when the experimental intervention represented the ICI strategy under evaluation and the comparator reflected the established standard of care. Dual-checkpoint blockade regimens consisting exclusively of immune checkpoint inhibitors were considered eligible and analyzed separately. Hazard ratios (HRs) for recurrence-related outcomes (DFS/RFS/PFS) and overall survival (OS) were pooled using random-effects models. A sensitivity analysis restricted to phase III trials was performed to evaluate the robustness of the primary findings. Subgroup analyses were conducted by tumor type and ICI mechanism of action.

**Results:**

Twenty-one randomized controlled trials (RCTs), including 17,446 randomized patients across eight different solid tumors, were analyzed. These trials contributed 23 treatment comparisons due to multiple experimental comparisons from IMMUNED and CheckMate 914. Adjuvant ICIs significantly improved DFS/RFS/PFS (HR 0.74, 95% CI 0.68–0.82, p<0.0001) and OS (HR 0.84, 95% CI 0.78–0.90, p<0.0001) compared with non-ICI controls. The DFS/RFS benefit was observed across several tumor types, including melanoma, urothelial carcinoma, renal cell carcinoma, and NSCLC (HRs 0.65, 0.78, 0.84, and 0.87, respectively). Regarding ICI class, significant DFS/RFS improvements were observed with CTLA-4, anti–PD-1, and anti–PD-L1 inhibitors (HRs 0.77, 0.69, and 0.89, respectively). Exploratory meta-regression identified a significant overall association between ICI class and treatment effect (QM p = 0.0183; R² = 45.2%); however, no individual checkpoint class showed a statistically significant difference compared with the reference category. These findings should therefore be interpreted cautiously and considered hypothesis-generating. Tumor-specific OS benefits were statistically significant in melanoma and urothelial carcinoma (HRs 0.76 and 0.85, respectively), whereas OS effects in other tumor types remain uncertain due to limited data availability and variable follow-up maturity.

**Discussion:**

Overall, these findings support the efficacy of adjuvant ICIs across multiple solid tumors, with consistent improvements in DFS/RFS and a significant pooled OS benefit. Exploratory meta-regression suggested that tumor type may contribute to between-study heterogeneity; however, results should be interpreted cautiously given the limited number of studies, trial-level differences, and potential ecological bias.

**Systematic review registration:**

https://www.crd.york.ac.uk/prospero/, identifier CRD420251249507.

## Introduction

In recent years, immunotherapy has profoundly reshaped the adjuvant treatment landscape, with immune checkpoint inhibitors (ICIs) demonstrating efficacy across several tumor types. The primary goal of this adjuvant strategy is to eradicate micrometastatic disease, thereby reducing the risk of recurrence and improving long-term survival. Landmark clinical trials have established the efficacy of this approach across several tumor types and have contributed to redefining the standard of care.

In melanoma, adjuvant immune checkpoint inhibitors have markedly improved outcomes in high-risk resected disease. Initial trials established the benefit of ipilimumab, while subsequent studies confirmed that PD-1 inhibitors (pembrolizumab and nivolumab) as standard of care across stage III and more recently stage IIB/C and resected stage IV melanoma, consistently demonstrating significant reductions in recurrence risk and durable survival benefit (1–7).

In non–small-cell lung cancer (NSCLC), adjuvant ICI therapy has become standard after surgery and chemotherapy in stage IB–IIIA disease. IMpower010 demonstrated improved disease-free survival (DFS) with atezolizumab, particularly in PD-L1–positive stage II–IIIA tumors, while PEARLS/KEYNOTE-091 confirmed a significant DFS benefit with pembrolizumab versus placebo, consolidating the role of anti–PD-1/PD-L1 therapy in early-stage NSCLC (8–11).

In urothelial carcinoma, adjuvant nivolumab significantly improved disease-free survival (DFS), particularly in PD-L1–positive patients, as shown in CheckMate 274. A DFS benefit was also observed with pembrolizumab in AMBASSADOR (Alliance A031501) trial, supporting a consistent effect of anti–PD-1 agents in this setting. In contrast, IMvigor010 did not demonstrate a benefit with adjuvant atezolizumab versus observation (12–14).

In high-risk renal cell carcinoma (RCC), KEYNOTE-564 demonstrated significant improvements in disease-free survival (DFS) and a subsequent overall survival (OS) benefit with adjuvant pembrolizumab. In contrast, IMmotion010 (atezolizumab) and CheckMate 914 (nivolumab-based regimens) failed to show a significant DFS advantage, highlighting inconsistent results across agents in this setting (15–18).

In gastrointestinal malignancies, CheckMate 577 established adjuvant nivolumab as a standard option for esophageal/GEJ cancers with residual disease after neoadjuvant therapy, demonstrating a significant disease-free survival (DFS) benefit. Other studies showed mixed results: durvalumab after chemoradiotherapy in ESCC did not meet all primary endpoints, and the VESTIGE trial failed to improve DFS in high-risk gastric/GEJ adenocarcinoma. In contrast, adjuvant sintilimab significantly improved DFS in resected high-risk hepatocellular carcinoma (19–22).

Early results of adjuvant tislelizumab for recurrent resected nasopharyngeal carcinoma showed a significant PFS benefit. In the context of high-risk resected locally advanced squamous cell carcinoma of the head and neck, the NIVOPOST-OP trial demonstrated that adding nivolumab to postoperative radiotherapy and concomitant cisplatin-based chemotherapy significantly improved DFS compared to standard care alone (23, 24).

However, the magnitude and consistency of the benefit of adjuvant ICIs across solid tumors, including differences by tumor histology, disease stage, and mechanism of action, remain incompletely defined.

## Methods

This meta-analysis of phase II and III randomized controlled trials aimed to evaluate the impact of adjuvant ICIs compared with non-ICI controls on disease-free survival (DFS) and overall survival (OS) in patients with resected solid tumors.

This meta-analysis was prospectively registered in PROSPERO (registration number: CRD420251249507). The protocol was updated to align the registered methods with the final review. The eligibility criteria were expanded to include both phase II and phase III randomized controlled trials. The literature search was updated to reflect all databases searched, and the risk of bias assessment was revised from Cochrane RoB-1 to the Cochrane Risk of Bias 2 (RoB 2) tool, in accordance with current methodological standards. These amendments did not alter the review objectives or primary outcomes.

A systematic literature search was conducted in PubMed, Embase, the Cochrane Central Register of Controlled Trials (CENTRAL), and the proceedings of the American Society of Clinical Oncology (ASCO) and the European Society for Medical Oncology (ESMO) to identify relevant studies published between January 2015 and June 2026. The search strategy included the following terms and their equivalents: (“immunotherapy” OR “immune checkpoint inhibitor” OR drug-specific terms such as pembrolizumab, nivolumab, atezolizumab, durvalumab, ipilimumab, or dostarlimab) AND (“adjuvant” OR “adjuvant therapy”) AND (“neoplasms” OR cancer* OR tumor* OR carcinoma*) AND (“randomized controlled trial” OR randomized OR randomised). The complete electronic search strategies for all bibliographic databases and conference proceedings (ASCO and ESMO) are provided in **Supplementary Table 1**. Eligible comparator arms included observation, placebo, standard therapy, chemotherapy, interferon-based therapy, or other active systemic treatments according to the original trial design. Trials comparing an ICI-containing adjuvant strategy with another active immunotherapy regimen were also considered eligible when the experimental arm represented the ICI strategy under investigation and the comparator reflected the established standard of care at the time of trial design. Eligible interventions included adjuvant ICI monotherapy as well as dual-checkpoint blockade regimens (e.g., nivolumab plus ipilimumab), provided that the randomized intervention consisted exclusively of immune checkpoint inhibition. Exclusion criteria comprised single-arm studies, concurrent combinations of ICIs with chemotherapy, radiotherapy, vaccines, targeted therapies, non-randomized studies, duplicate publications, and studies involving neoadjuvant immune checkpoint inhibitor therapy, as this analysis was strictly limited to the postoperative adjuvant setting. Studies in which patients had received prior chemotherapy or chemoradiotherapy before randomization were considered eligible if the randomized comparison evaluated postoperative adjuvant ICI versus a non-ICI control. Hazard ratios (HRs) for DFS/RFS and OS were extracted preferentially from the intention-to-treat (ITT) population. When multiple analyses were reported within a trial, ITT estimates were selected to ensure consistency across studies and to minimize selection bias.

Data extraction and quality assessment were independently conducted by two reviewers, with discrepancies resolved by consensus or by a third reviewer. From each eligible trial, information on study design, population characteristics, treatment regimens, follow-up duration, and reported outcomes was collected. The risk of bias of the included randomized controlled trials was assessed using the Cochrane Risk of Bias 2 (ROB2) tool, evaluating the following domains: randomization process; deviations from intended interventions; missing outcome data; measurement of the outcome; and selection of the reported results. An overall risk of bias judgment was assigned for each study according to ROB2 guidance (**Supplementary Figure 1**; **Supplementary Table 2**).

**Figure 1:**
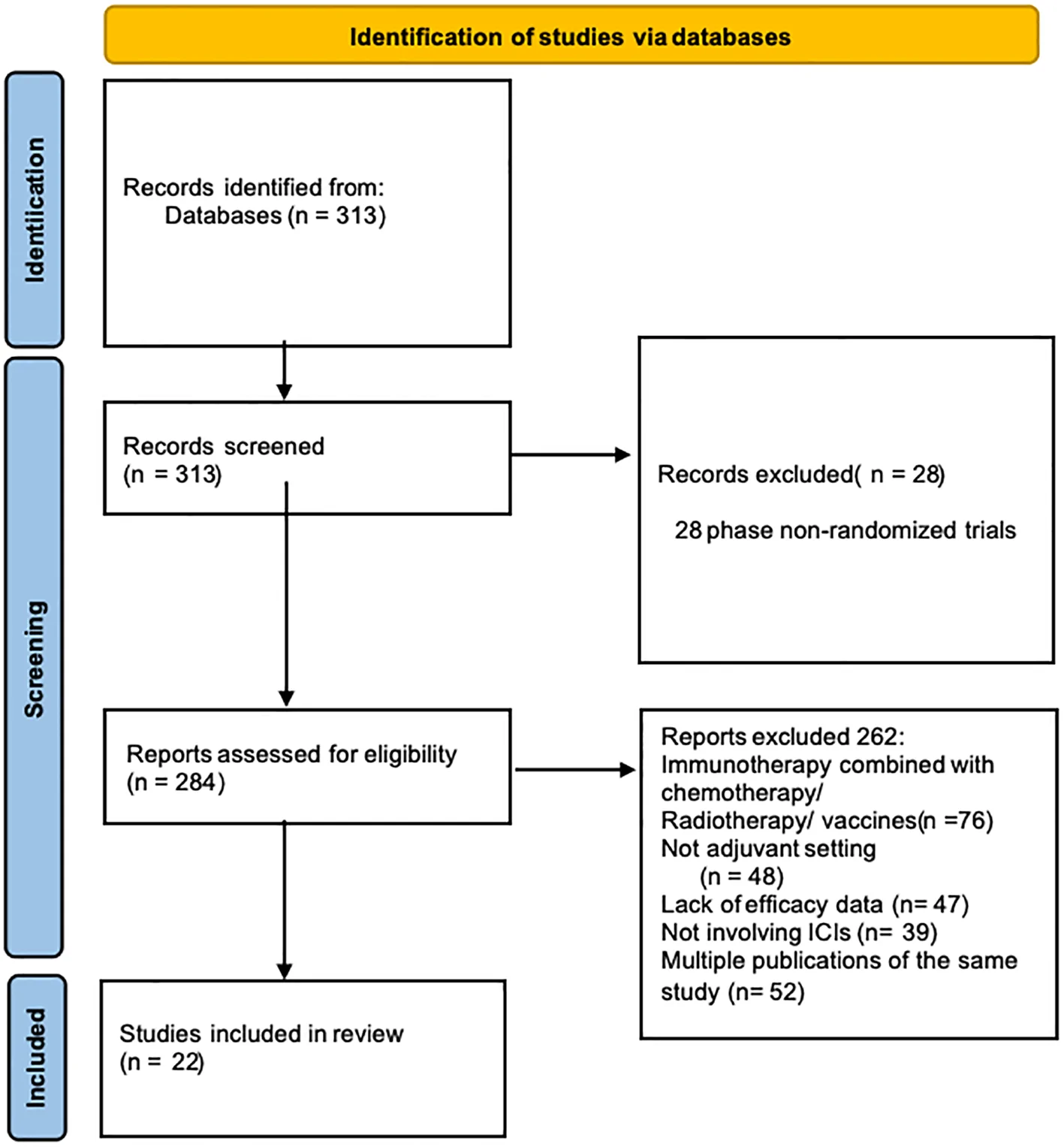
PRISMA flow diagram illustrating the selection process of studies included in the systematic review and meta-analysis.

This study was performed in accordance with the Preferred Reporting Items for Systematic Reviews and Meta-Analyses (PRISMA) statement (**Figure 1**).

A meta-analysis of efficacy data was also performed, and hazard ratios (HRs) were pooled for disease-free survival and overall survival. All analyses were conducted using a random-effects model, and quantitative between-trial heterogeneity (I²) was variably present across comparisons. DFS and RFS were considered the primary recurrence-related endpoints. Because one eligible randomized trial reported PFS as the only available recurrence-related time-to-event outcome in the postoperative setting, PFS was included in the primary pooled analysis as a surrogate measure of disease control. A sensitivity analysis excluding this study was performed to evaluate the impact of endpoint heterogeneity on the pooled estimate. DFS and RFS were considered comparable recurrence endpoints; however, RFS was predominantly reported in melanoma trials, and therefore both endpoints were analyzed together in the primary analysis, with sensitivity analyses accounting for endpoint definition differences. A sensitivity analysis excluding the study reporting PFS as the primary endpoint (23) was also performed to evaluate the impact of endpoint heterogeneity on the pooled estimate. To avoid unit-of-analysis errors arising from shared control groups, only the monotherapy comparison was included in the primary pooled analysis of DFS/OS. Dual-checkpoint blockade comparisons sharing the same placebo arm were analyzed separately. Heterogeneity among studies was assessed using the I² statistic. A random-effects model was employed to pool the hazard ratios to account for between-study variability. However, to account for potential differences in endpoint definitions, sensitivity analyses were subsequently performed separating DFS and RFS outcomes. The same random-effects model was applied to all supplementary sensitivity analyses, including analyses stratified by endpoint definition and follow-up maturity.

In addition, sensitivity analyses restricted to phase III trials were performed to evaluate the robustness of the primary results and to assess whether inclusion of phase II studies influenced pooled effect estimates.

**Figure 2:**
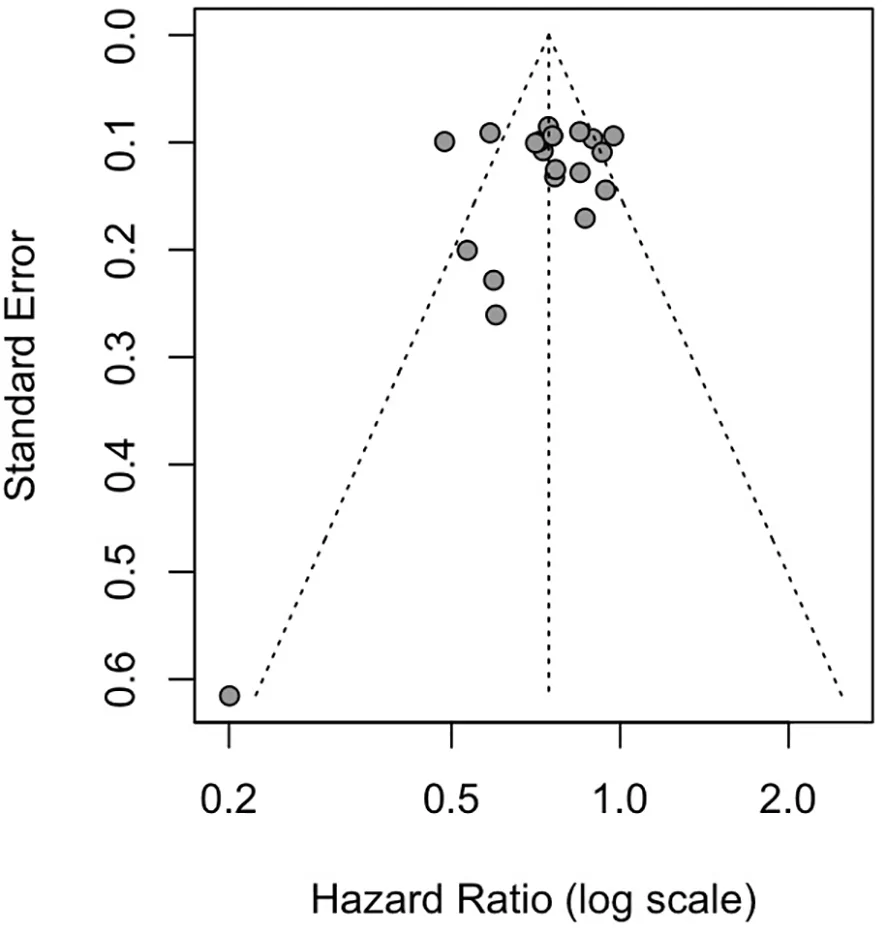
Funnel plot assessing publication bias for the pooled analysis of disease-free survival in patients receiving adjuvant ICIs.

Funnel plots were used to assess publication bias and Egger’s test was performed (**Figure 2**). Funnel plot asymmetry was interpreted with caution, as it may reflect clinical heterogeneity or other small-study effects rather than publication bias alone. The R language (R Foundation for Statistical computing, version 4.4.2) was used for the meta-analysis.

Meta-regression analyses were performed to explore sources of between-study heterogeneity.

This analysis provides an updated evaluation of adjuvant immune checkpoint inhibitors across multiple solid tumors, while acknowledging the biological and clinical differences that may limit direct comparability between tumor types.

## Results

This systematic review and meta-analysis, including 21 phase II/III randomized controlled trials and 17,446 randomized patients, provides comprehensive evidence supporting the use of adjuvant immune checkpoint inhibitors across a broad spectrum of solid tumors. The main characteristics of the included randomized clinical trials are summarized in **Table 1**. The included studies evaluated multiple classes of ICIs, predominantly anti–PD-1 agents, with additional trials investigating anti–PD-L1 and anti–CTLA-4 therapies. Most studies evaluated adjuvant ICI monotherapy, whereas three treatment comparisons assessed dual-checkpoint blockade with nivolumab plus ipilimumab. Several trials allowed prior chemotherapy or chemoradiotherapy before randomization; however, in all included studies the randomized intervention consisted exclusively of postoperative immune checkpoint inhibition, either as monotherapy or as dual-checkpoint blockade, compared with a non-ICI control.

**Table 1:** Main characteristics of randomized clinical trials evaluating adjuvant immune checkpoint inhibitors (ICIs) in resected solid tumors.

Primary tumor	Source publication (trial)	Trial phase	Stage (N experimental vs control arm)	Randomized comparison	Prior adjuvant systemic treatment	HR DFS / RFS	PD-L1 assessment (cut-off)	HR DFS/RFS PD-L1 positive	Primary endpoint	Duration (years)	Median follow-up (data cut-off)	OS maturity/ deaths (experimental arm)	Crossover	Subsequent therapy
Melanoma	Eggermont et al., 2019 (1)(EORTC 18071)	III	III Resected (475 Vs 476)	Ipilimumab Vs Placebo	NA	0.75 (ITT Population)	–	–	RFS	3	63.6 mos (31 Jan 2016)	Mature HR 0.73 Deaths: 173	Allowed	Chemoterapy 31.1%, Surgery 39.6%; BRAF inhibitor 25.6%
	Eggermont et al., 2021 (3)(KEYNOTE-054)	III	III Resected (514 Vs 505)	Pembrolizumab Vs Placebo	NA	0.59 (ITT Population)	IHC (≥2)	0.59	RFS	1	42.3 mos (03 Apr 2020)	Mature HR 0.60	Allowed	Unknown
	Yoon et al. 2025 (7) (KEYNOTE-716)	III	IIB/IIC Resected (487 Vs 489)	Pembrolizumab Vs Placebo	NA	0.60 (ITT Population)	–	–	RFS	1	52.8 mos (16 Feb 2024)	NR	Not allowed	Unknown
	Kirkwood et al., 2023 (5)(CheckMate 76K)	III	IIB/IIC Resected (526 Vs 264)	Nivolumab Vs Placebo	NA	0.42 (ITT Population)	–	–	RFS	1	15.8 mos (28 Jun 2022)	NR	Not allowed	Surgery 6.8%; systemic Therapy 5.7%
	Livingstone et al., 2022 (6)(IMMUNED)	II	Resected Stage IV (NED) (59 Vs 52)	Nivolumab Vs Placebo	NA	0.60 (ITT Population)	–	–	RFS	1	49.2 mos (23 Sept 2021)	Mature HR 0.41 Deaths: 22% at 49.2 mos	Allowed	Target therapy n=13; Immunotherapy n= 11
	Livingstone et al., 2022 (6)(IMMUNED)	II	Resected Stage IV (NED) (56 Vs 52)	Nivolumab + Ipilimumab Vs Placebo	NA	0.25 (ITT Population)	–	–	RFS	1	49.2 mos(23 Sept 2021)	Mature HR 0.75 Deaths: 13% at 49.2 mos	Allowed	Target therapy n=4; Immunotherapy n= 7
	Grossman et al., 2024 (4) (SWOG S1404)	III	IIIA(N2a)/IIIB/IIIC/IV Resected (648 Vs 655)	Pembrolizumab Vs Interferon-alfa 2b/ Ipilimumab	NA	0.62 (ITT Population)	–	–	RFS	1	47.5 mos	Mature HR 0.82	Not allowed	Unknown
	Tarhini et al., 2019 (2) (E1609)	III	IIIB/IIIC/M1a-b Resected (561 Vs 635)	Ipilimumab Vs Interferon-alfa 2b	NA	0.85 (ITT Population)	–	–	RFS	1	75.4 mos (15 Feb 2019)	Mature HR 0.78 Deaths: 264	Not allowed	Unknown
NSCLC	Felip et al., 2025 (8) (IMpower010)	III	IB–IIIA Resected (507 Vs 498)	Atezolizumab Vs Observation	Platinum-based chemotherapy	0.85 (ITT Population)	TC expression (≥1%)	0.70	DFS	1	32.2 mos (21 Jan 2021)	Mature HR 0.77 Deaths: 156	Not allowed	Unknown
	O’Brien et al., 2022 (9)(KEYNOTE-091/PEARLS)	III	IB–IIIA Resected (590 Vs 587)	Pembrolizumab Vs Placebo	Optional adjuvant chemotherapy	0.76 (ITT Population)	TPS (≥50%)	0.82	DFS	1	35.6 mos (20 Sept 2021)	Mature HR 0.76 Deaths: 18% at 36 mos	Not allowed	Unknown
	Chaft et al., 2026 (10)	III	IB–IIIA Resected without sensitizing sequence variants in EGFR and ALK (466 Vs 469)	Nivolumab Vs Observation	Optional adjuvant chemotherapy	0.97 (ITT Population)	TC expression ≥50%	0.86	DFS	1	72.6 mos (Dec 2025)	Mature HR 1.02	Not allowed	Unknown
	Goss et al., 2026 (11)	III	IB–IIIA Resected (815 Vs 404)	Durvalumab Vs Placebo	Optional adjuvant chemotherapy	0.93 (ITT Population)	–	–	DFS	1	60 mos (10 Nov 2022)	NR	Not allowed	Unknown
Urothelial carcinoma	Galsky et al., 2024 (12) (CheckMate 274)	III	High Risk MIUC (post-cystectomy) (353 Vs 356)	Nivolumab Vs Placebo	NA	0.71 (ITT Population)	IHC 28-8 pharmDx assay (≥1%)	0.52	DFS	1	36.1 mos	Mature HR 0.56 Deaths: 35% at 36 mos	Not allowed	Unknown
	Bellmunt et al., 2021 (14) (IMvigor010)	III	MIUC (post-cystectomy) (406 Vs 403)	Atezolizumab Vs Observation	NA	0.89 (ITT Population)	–	–	DFS	1	21.9 mos (30 Nov 2019)	Mature HR 0.85 Deaths: 114	Not allowed	Unknown
	Apolo et al., 2025 (13)(AMBASSADOR/ Alliance A031501)	III	High Risk (post-cystectomy) (354 Vs 348)	Pembrolizumab Vs Observation	NA	0.73 (ITT Population)	CPS (≥10%)	0.81	DFS	1	44 mos (5 Jul 2024)	Mature HR 0.98 Deaths: 257	Not allowed	Antibody-drug conjugates 34.2%; Chemotherapy in 26.7%, ICIs 15.1%
RCC	Choueiri et al. (KEYNOTE-564)	III	Localized (High Risk) (496 Vs 498)	Pembrolizumab Vs Placebo	NA	0.72 (ITT Population)	–	–	DFS	1	57.2 mos (15 Sept 2023)	Mature HR 0.62 Deaths: 132	Not allowed	Systemic an ticancer drug therapy 79.5%; Surgery 27.3%
	Motzer et al., 2024 (18) (CheckMate 914 Part B)	III	Localized (High Risk) (411 Vs 208)	Nivolumab Vs Placebo	NA	0.87 (ITT Population)	IHC 28-8 pharmDx assay (≥1%)	0.45	DFS	1	37 mos (28 Jun 2022)	NR	Not allowed	VEGF-targeted agent 11.2%
	Motzer et al., 2024 (17) (CheckMate 914 Part A)	III	Localized (High Risk) (405 Vs 411)	Nivolumab+ Ipilimumab Vs Placebo	NA	0.92 (ITT Population)	–	–	DFS	1	37 mos (28 Jun 2022)	NR	Not allowed	VEGF-targeted agent 12.6%
	Kumar Pal et al. 2022 (16)(IMmotion010)	III	Localized (Increased Risk of Recurrence After Resection) (390 Vs 388)	Atezolizumab Vs Placebo	NA	0.93 (ITT Population)	–	–	DFS	1	44.7 mos (3 May 2022)	Mature HR 0.97 Deaths: 107	Not allowed	TKI 22%; ICIs 11%
Gastrointestinal Cancer	Kelly et al., 2021 (19)(CheckMate 577)	III	ESCC or GEJ Cancer, stage II or III Resected after neoadjuvant CTRT with residual pathological disease (532 Vs 262)	Nivolumab Vs Placebo	NA	0.76 (ITT Population)	–	–	DFS	1	24.4 mos (12 May 2020)	Mature Adenocarcinoma HR 0.75; Squamous HR 0.61 Deaths:396	Not allowed	Systemic therapy 23%; Radiotherapy 8%; Surgery 5%
	Lordick et al., 2025 (21)(EORTC 1707 VESTIGE)	II	Gastric and GEJ adenocarcinoma Resected following neoadjuvant CT (high risk: ypN+ and/or R1) (98 Vs 97)	Nivolumab+ Ipilimumab Vs Physician's choice CT	NA	1.55 (ITT Population)	–	–	DFS	1	25.3 mos (12 Dec 2023)	Mature HR 1.32 Deaths: 73	Not allowed	Unknown
	Wang et al., 2024 (22)	II	Resected High-Risk HCC (with microvascular invasion) (99 Vs 99)	Sintilimab Vs Observation	NA	0.53 (ITT Population)	–	–	RFS	8 cycles	23.3 mos (1 May 2023)	Mature HR 0.50 Deaths = 12	Not allowed	Unknown
Nasopharyngeal Carcinoma	Li et al., 2025 (23)	II	Adjuvant, following endoscopic surgery (22 Vs 20)	Tislelizumab Vs Observation	NA	0.19 (ITT Population)	–	–	PFS	1	18 mos (4 Nov 2024)	Mature HR 0.31 Deaths: 1	Not allowed	Unknown

Mos, Months; MIUC, muscle-invasive urothelial carcinoma; DFS, Disease-Free Survival; RFS, Recurrence-Free Survival; PFS, Progression-Free Survival; CT, chemotherapy; CTRT, chemioradiotherapy; TC, Tumor cell; NR, Not Reached; ITT, Intention-to-treat.

Patient populations ranged from stage I to stage IV disease, and control groups included placebo, observation, chemotherapy, or standard care. Despite heterogeneity in tumor types, disease stages, and treatment regimens, this comprehensive dataset collectively reflects the expanding role of ICIs as adjuvant therapy across early-stage solid tumors.

**Figure 3:**
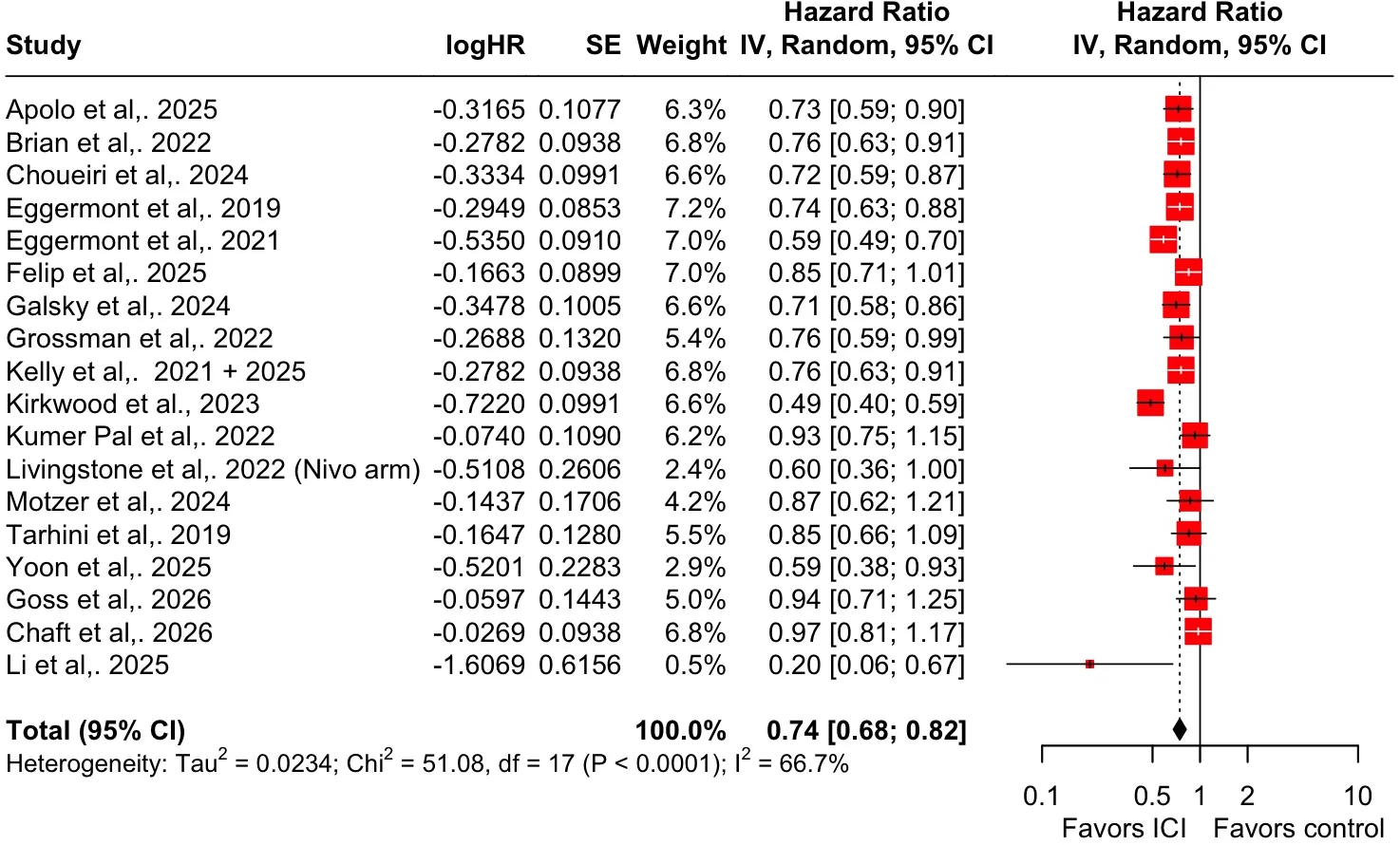
Forest plot of pooled recurrence-related outcomes (DFS/RFS/PFS) for patients treated with adjuvant immune checkpoint inhibitors (ICIs) versus randomized comparator arms across solid tumors.

**Figure 4:**
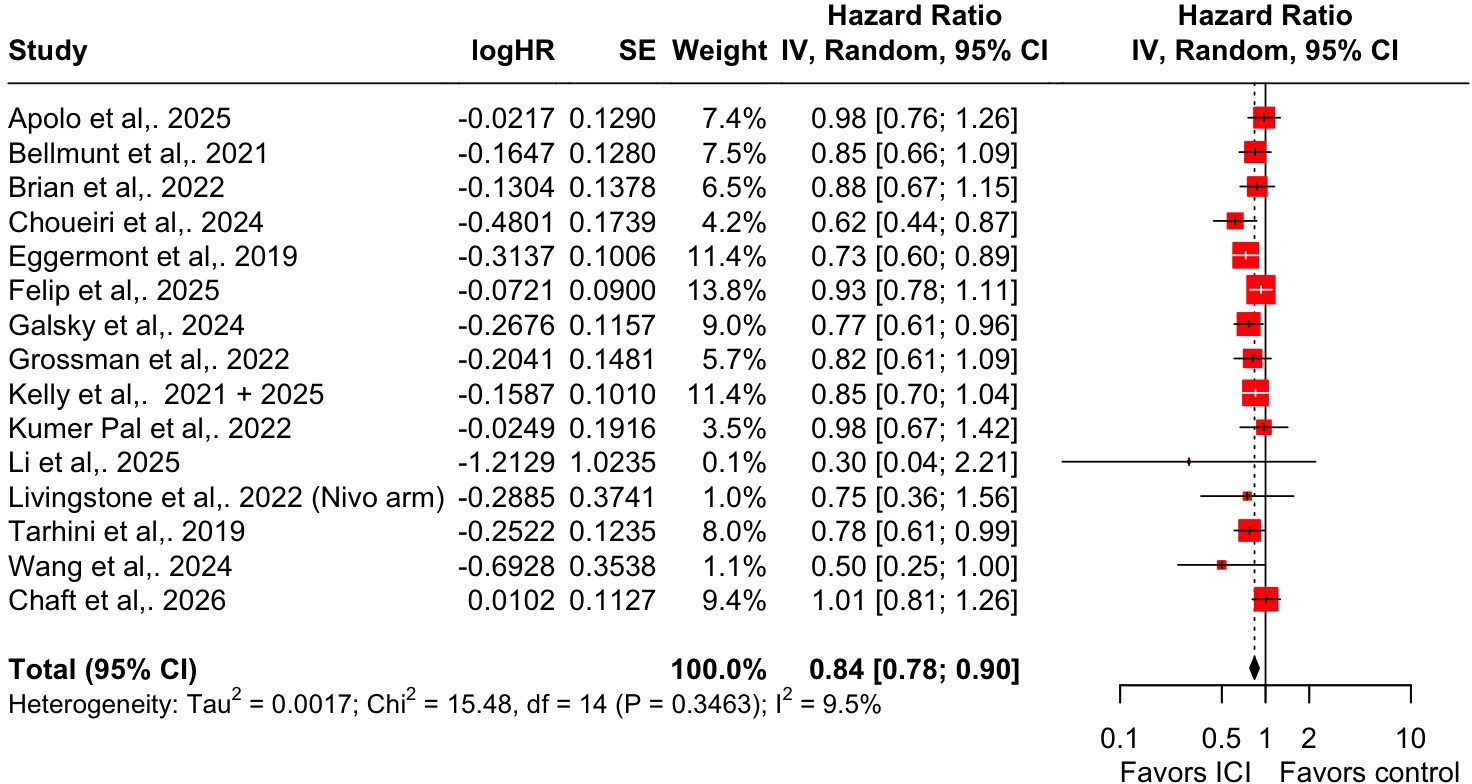
Forest plot of pooled overall survival (OS) for all patients treated with adjuvant ICIs versus non-ICI controls across solid tumors.

Adjuvant immune checkpoint inhibitors significantly improved disease-free survival/recurrence-free survival (DFS/RFS/PFS; analyzed in the ITT population) (HR 0.74, 95% CI 0.68–0.82; I² = 66.7%; p < 0.0001) and overall survival (OS) (HR 0.84, 95% CI 0.78–0.90; I² = 9.5%; p < 0.0001) compared with non-ICI controls across all solid tumors (**Figures 3**, **4**). To assess the potential impact of trial phase, sensitivity analyses restricted to phase III studies were performed. The DFS/RFS benefit remained essentially unchanged (HR 0.76, 95% CI 0.70–0.83; I² = 67.3%; p < 0.0001), and the OS benefit was similarly confirmed (HR 0.85, 95% CI 0.79–0.91; I² = 9.4%; p < 0.0001) (**Supplementary Figure 2**).

**Figure 5:**
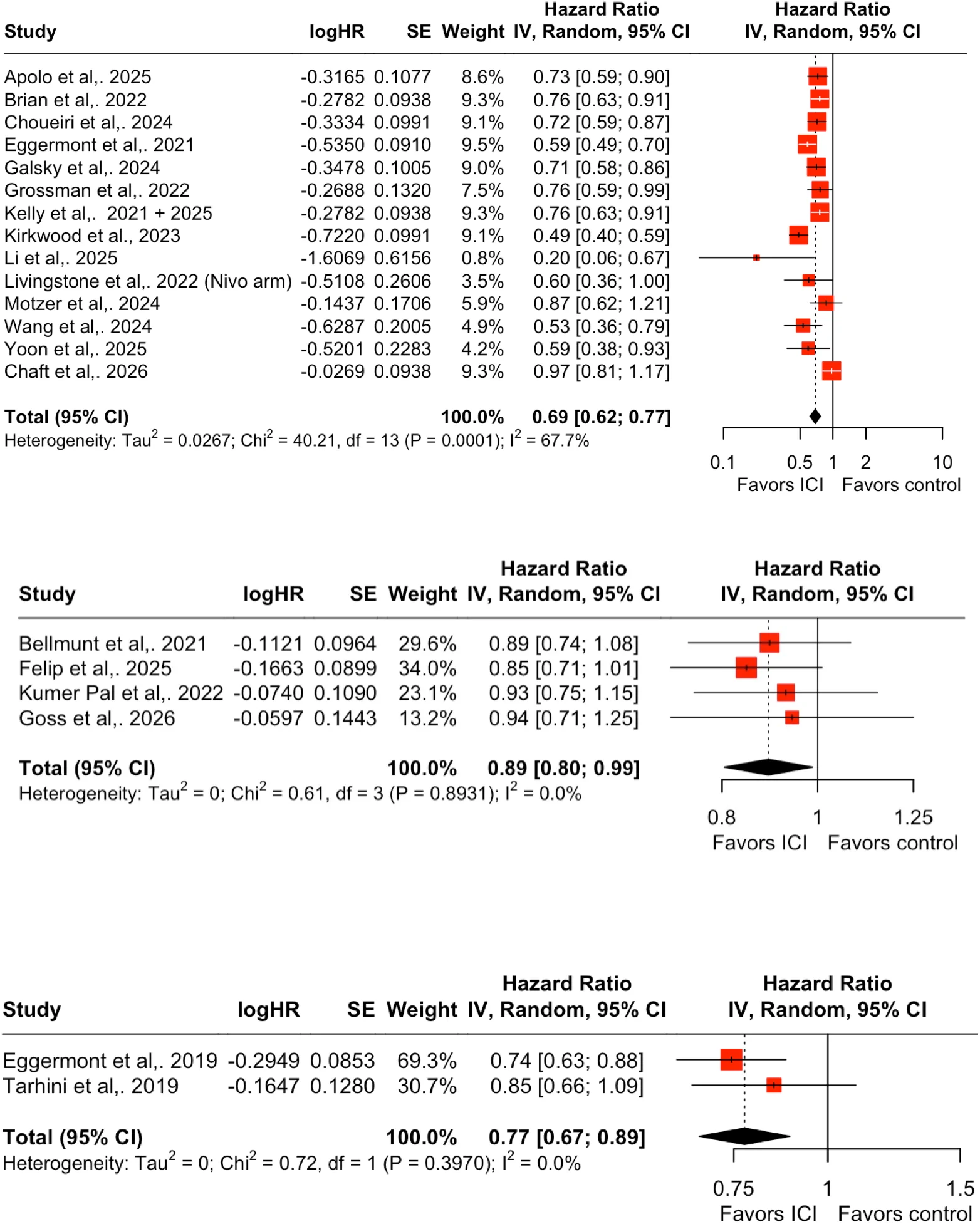
Subgroup analysis according to immune checkpoint target. Forest plots showing the effect of PD-1 inhibitors (upper panel), PD-L1 inhibitors (middle panel), and CTLA-4 inhibitors (lower panel) on disease-free survival (DFS).

Across immune checkpoint mechanisms, CTLA-4 inhibitors, PD-1 inhibitors, and PD-L1 inhibitors all demonstrated clinically meaningful reductions in recurrence risk. CTLA-4 blockade significantly improved DFS (HR 0.77, 95% CI 0.67–0.89; I² = 0.0%; p = 0.0003) (Lower panel, **Figure 5**). PD-1 inhibitors were associated with a statistically significant improvement in DFS/RFS (HR 0.69, 95% CI 0.62–0.77; I² = 67.7%; p < 0.0001), whereas PD-L1 inhibitors also demonstrated a statistically significant benefit (HR 0.89, 95% CI 0.80–0.99; I² = 84.7%; p = 0.029).(Middle panel, **Figure 5**).

**Figure 6:**
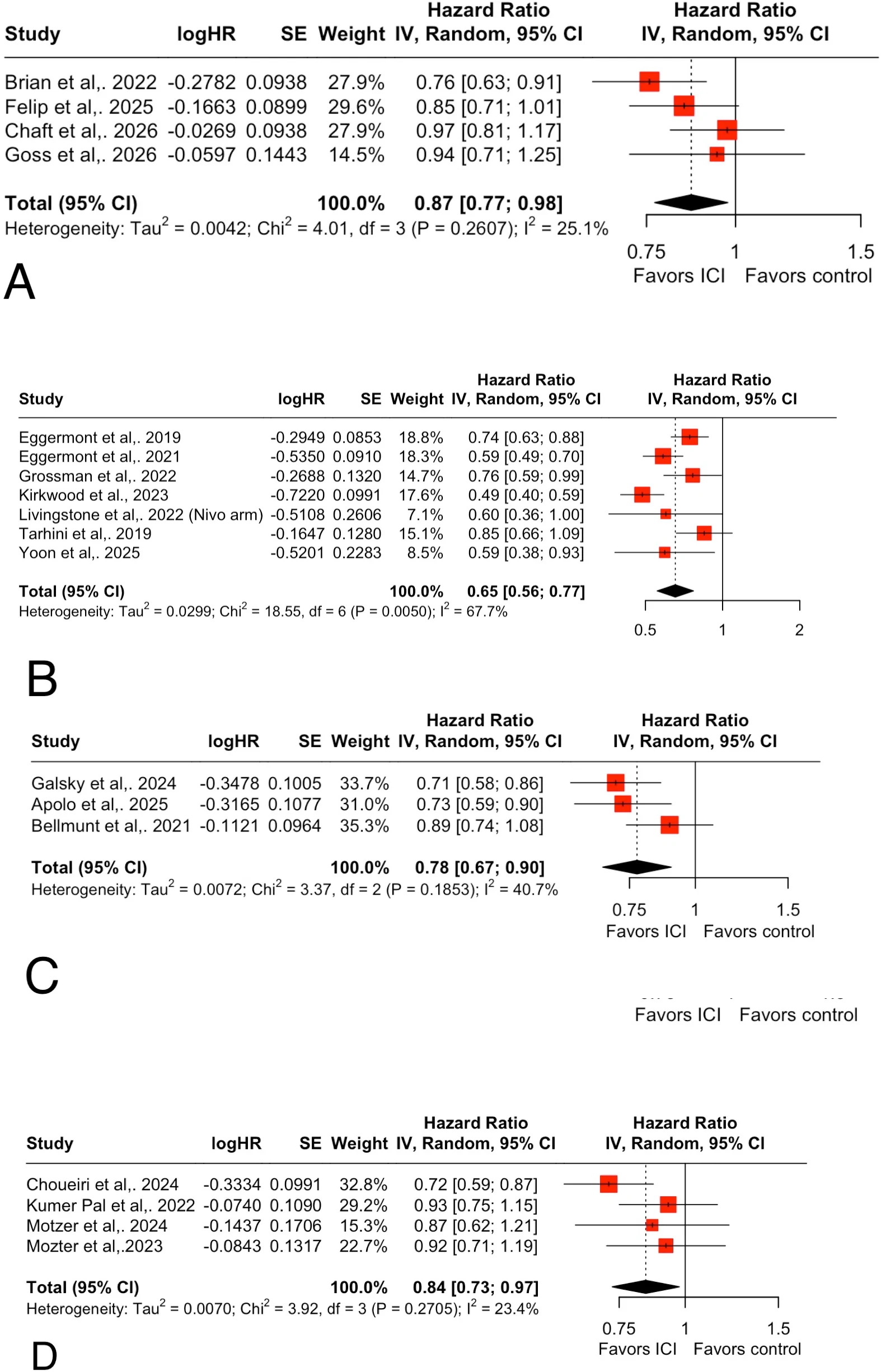
Subgroup analysis of disease-free survival (DFS) according to primary tumor type. Forest plots are shown for **(A)** non–small-cell lung cancer (NSCLC), **(B)** melanoma,**(C)** urothelial carcinoma, and **(D)** renal cell carcinoma (RCC).

When examined across different primary tumor types, the magnitude of DFS/RFS improvement varied. Melanoma showed a significant RFS improvement (HR 0.65, 95% CI 0.56–0.77; I² = 67.7%; p < 0.0001). Significant DFS/RFS benefits were also observed in non–small-cell lung cancer (HR 0.87, 95% CI 0.77–0.98; I² = 25.1%; p = 0.018) and urothelial carcinoma (HR 0.78, 95% CI 0.67–0.90; I² = 40.7%; p = 0.0009). In RCC, adjuvant ICIs were associated with a statistically significant improvement in disease-free survival (DFS) (HR 0.84, 95% CI 0.73–0.97; I² = 23.4%; p = 0.021) (**Figure 6**).

In sensitivity analyses, separating studies according to the recurrence endpoint confirmed the robustness of the findings; DFS remained significantly improved (HR 0.82, 95% CI 0.76–0.88; p < 0.0001) with low heterogeneity (I² = 24.8%), whereas RFS showed an even stronger benefit (HR 0.64, 95% CI 0.55–0.75; p < 0.0001) but with higher between-study heterogeneity (I² = 64.0%) (**Supplementary Figure 3**).

In subgroup analysis according to control arm, adjuvant immune checkpoint inhibitors demonstrated a significant DFS/RFS benefit in both placebo-controlled trials (HR 0.71, 95% CI 0.64–0.80; p < 0.0001; I² = 66.3%) and observation-controlled trials (HR 0.79, 95% CI 0.67–0.94; p = 0.0070; I² = 67.0%), with a consistent effect across study designs despite moderate heterogeneity. (**Supplementary Figure 4**) Owing to the limited number of studies using interferon, chemotherapy or active immunotherapy comparators, separate pooled analyses for these comparator categories were not considered statistically meaningful.

Treatment sequencing was explored descriptively according to whether ICIs were administered after surgery alone or following previous chemotherapy/chemoradiotherapy. However, the limited number of trials within each sequence category and the strong correlation between treatment sequence and tumor type precluded reliable comparative meta-analysis. Therefore, sequence-specific effects should be considered hypothesis-generating.

Furthermore, death counts or overall survival (OS) event maturity, subsequent therapies, and crossover information were insufficiently reported or largely unavailable across the included trials; consequently, these factors could not be formally analyzed or used to draw definitive conclusions, and their potential influence on the pooled OS estimate must be acknowledged.

Regarding overall survival (OS), adjuvant immune checkpoint inhibitors demonstrated meaningful clinical benefits across mechanisms and tumor types (1–3, 12, 15, 25).

**Figure 7:**
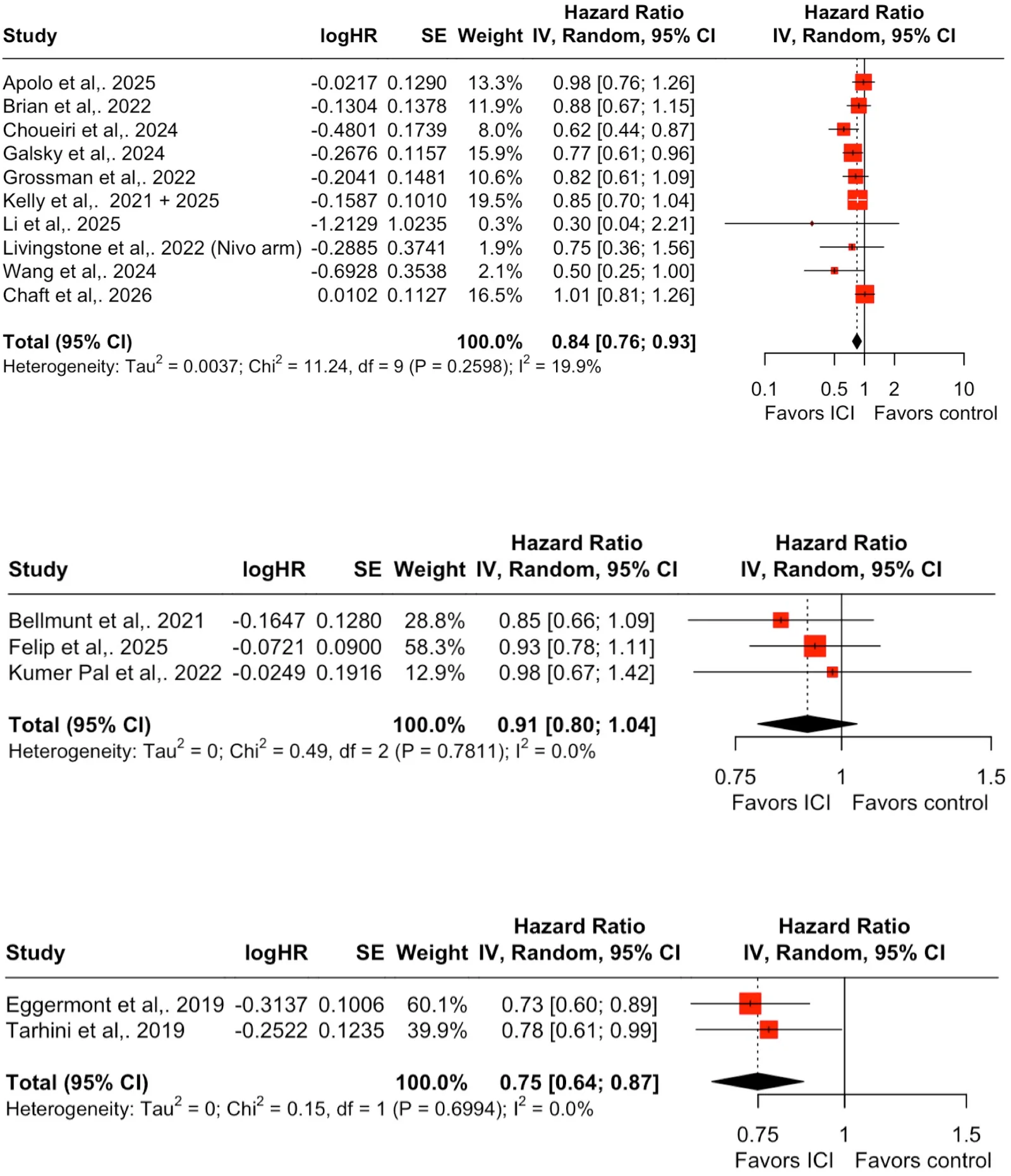
Subgroup analysis according to immune checkpoint target. Forest plots showing the effect of PD-1 inhibitors (upper panel), PD-L1 inhibitors (middle panel), and CTLA-4 inhibitors (lower panel) on Overall survival (OS).

Similarly, PD-1 inhibitors showed a statistically significant OS benefit (HR 0.84, 95% CI 0.76–0.93; I² = 19.9%; p < 0.0001), whereas PD-L1 inhibitors did not achieve a statistically significant OS advantage (HR 0.91, 95% CI 0.80–1.04) (**Figure 7**).

**Figure 8:**
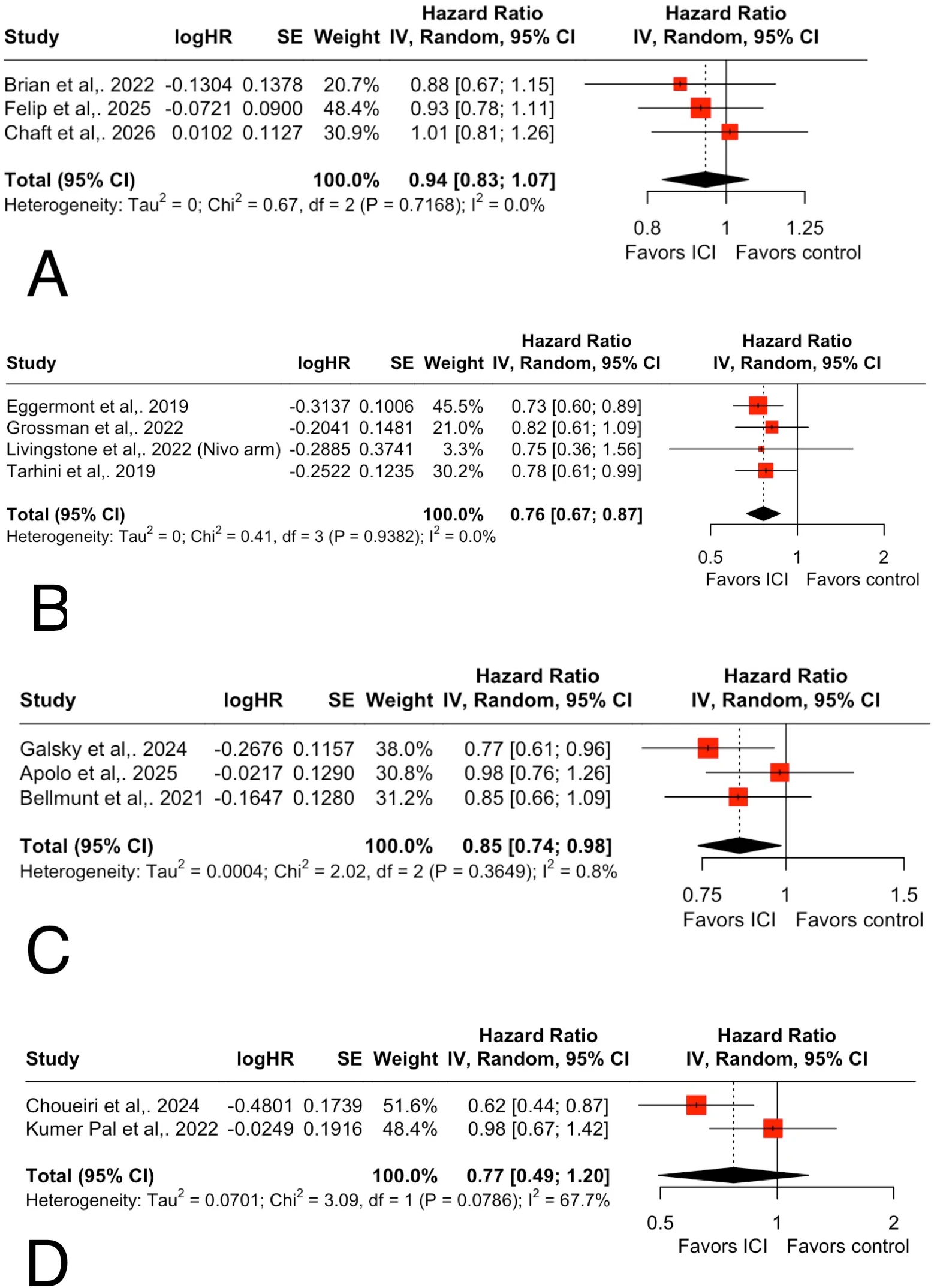
Subgroup analysis of Overall survival (OS) according to primary tumor type. Forest plots are shown for **(A)** non–small-cell lung cancer (NSCLC), **(B)** melanoma, **(C)** urothelial carcinoma, and **(D)** renal cell carcinoma (RCC).

By tumor type, melanoma patients experienced significant OS improvement (HR 0.76, 95% CI 0.67–0.87; I² = 0.0%; p =< 0.0001), as did those with urothelial carcinoma (HR 0.85, 95% CI 0.74–0.98; I² = 0.8%; p = 0.028). In contrast, non–small-cell lung cancer and RCC did not show a statistically significant OS benefit, (HR 0.94, 95% CI 0.81–1.07; HR 0.77, 95% CI 0.49-1.29) (**Figure 8**), suggesting heterogeneity in long-term survival outcomes across tumor types.

To address concerns regarding variability in follow-up duration and event maturity across trials, a sensitivity analysis of OS was performed restricting the pooled estimate to trials with a median follow-up of ≥24 months (k = 12). The maturity of OS data was assessed based on the availability of reported death events and median follow-up duration. However, death counts, OS event maturity, subsequent anticancer therapies, and crossover information were not consistently reported across trials. Therefore, these factors could not be incorporated into quantitative analyses and may have influenced the pooled OS estimates.

The OS benefit remained statistically significant and consistent with the primary analysis (HR 0.85, 95% CI 0.79–0.91; p < 0.0001), with low heterogeneity (I² = 9.4%). This finding supports the robustness of the overall survival benefit among trials with more mature follow-up (**Supplementary Figure 5**).

A separate pooled analysis was performed for trials evaluating dual-checkpoint blockade. The pooled estimate did not show a statistically significant benefit (HR 0.73, 95% CI 0.26–2.05; p = 0.55), and heterogeneity was very high (I² = 91.2%, 95% CI 77.3–96.6%; Q(df=2) = 22.82, p < 0.0001), reflecting substantial variability (**Supplementary Figure 6**). Given the small number of trials and the marked heterogeneity, this result should be interpreted with caution and considered exploratory rather than conclusive.

To investigate potential sources of between-study heterogeneity, an exploratory meta-regression analysis was performed using primary tumor type and selected clinical and demographic variables as moderators. In the model including primary tumor type as a categorical moderator (k = 24), the overall test of moderators was statistically significant (QM(df = 8) = 27.03, p = 0.0007), explaining a substantial proportion of the observed heterogeneity (R² = 71.67%). Residual heterogeneity was no longer statistically significant (QE(df = 15) = 18.65, p = 0.23), with a residual I² of 26.2%. Melanoma was used as the reference category. Significantly larger effect estimates were observed for gastric cancer (coefficient = 0.91, 95% CI 0.50–1.32, p < 0.0001), NSCLC (coefficient = 0.23, 95% CI 0.07–0.39, p = 0.004), and renal cell carcinoma (coefficient = 0.21, 95% CI 0.03–0.38, p = 0.019). No statistically significant differences were identified for the other tumor types. Separate univariable meta-regression models were subsequently fitted for ethnicity (coefficient = 0.001, p = 0.83), ECOG performance status (coefficient = −0.003, p = 0.28), treatment duration (all p values ranging from 0.47 to 0.97), and age (coefficient = 0.0003, p = 0.35). An exploratory meta-regression assessing ICI class as a categorical moderator showed a significant overall effect (QM(df = 3) = 10.03, p = 0.0183; R² = 45.2%), although no individual checkpoint class differed significantly from the reference category (anti-PD-1: p = 0.469; anti-PD-1 plus anti-CTLA-4: p = 0.107; anti-PD-L1: p = 0.393).

Given the limited number of included trials relative to the number of covariates examined, these meta-regression findings should be regarded as hypothesis-generating rather than confirmatory, and are discussed further as a study limitation below.

Publication bias was assessed using Egger’s linear regression test of funnel plot asymmetry. No statistically significant asymmetry was detected (t = 0.09, df = 16, bias estimate 0.15, SE = 1.61; p = 0.928), suggesting no evidence of significant publication bias among the included trials.

## Discussion

Previous pan-tumor meta-analyses have evaluated the efficacy of adjuvant PD-1/PD-L1 inhibitors across solid tumors, generally reporting improvements in disease-free survival based on approximately 10–15 randomized controlled trials. However, the current analysis expands the available evidence by incorporating 21 phase II–III randomized trials and 17,446 randomized patients, including not only PD-1/PD-L1 inhibitors but also CTLA-4–based and dual-checkpoint blockade strategies, as well as more recently published trials with extended follow-up. This updated evidence base provides a broader assessment of adjuvant immune checkpoint inhibition across multiple tumor types and allows an exploratory evaluation of potential sources of heterogeneity, including tumor characteristics and ICI class (25–28).

In the present meta-analysis, adjuvant immune checkpoint inhibitors were associated with improved recurrence-related outcomes across several solid tumor types, including melanoma, urothelial carcinoma, renal cell carcinoma, and NSCLC.

Significant tumor-specific OS benefits were observed in melanoma and urothelial carcinoma, whereas OS effects in other tumor types remain uncertain due to limited available data and variable follow-up maturity. However, these findings should be interpreted in the context of substantial heterogeneity across tumor entities and should not be considered as evidence of differential efficacy between tumor types without direct statistical comparison.

Significant DFS benefit was observed with CTLA-4-, PD-1-, and PD-L1-based regimens. Although exploratory meta-regression suggested that ICI class may contribute to between-study variability (QM p = 0.0183; R² = 45.2%), no individual checkpoint class demonstrated a statistically significant difference compared with the reference category. Therefore, differences between ICI subclasses should be considered hypothesis-generating rather than evidence of differential efficacy.

PD-L1 inhibitors were also associated with a statistically significant DFS benefit; however, this estimate was derived from a limited number of studies and should therefore be interpreted cautiously. Pairwise comparisons between individual ICI subclasses were not prespecified and were not performed; therefore, differences in efficacy estimates across checkpoint classes should be considered exploratory.

Improvements in DFS/RFS and OS were less pronounced, or not statistically significant, in renal cell carcinoma and non–small-cell lung cancer for certain endpoints, highlighting potential biological heterogeneity and the need for further investigation to optimize patient selection. Notably, the included trials were highly heterogeneous in tumor type, primary site, disease stage, immunotherapy regimen, and treatment duration, which may introduce potential biases and limit direct comparability.

The efficacy of dual-checkpoint blockade remains of considerable interest but could not be robustly addressed by the present analysis. Pooling of the three available treatment comparisons did not show a statistically significant benefit in DFS. This finding should not be interpreted as evidence against the efficacy of dual-checkpoint blockade, but rather highlights the current insufficiency of comparative data to draw firm conclusions on this question.

Meta-regression identified primary tumor type as a significant source of heterogeneity (QM p = 0.0007; R² = 71.7%), with gastric cancer, NSCLC, and renal cell carcinoma showing significantly different treatment effects relative to the reference category. Similarly, an exploratory meta-regression evaluating ICI class as a moderator showed a statistically significant overall effect, although no individual checkpoint category reached statistical significance. Given the limited number of studies and the risk of model instability, these findings should be interpreted cautiously and require validation in larger datasets. Other factors, including age, sex, race, duration of treatment and ECOG performance status, were not associated with treatment effect.

All analyses were conducted using a random-effects model, and quantitative between-trial heterogeneity (I²) was variably present across comparisons.

The meta-regression results should nonetheless be interpreted with caution and considered exploratory. The relatively small number of included trials, combined with the number of covariates examined, limits statistical power and increases the risk of model instability. Furthermore, because these analyses are based on study-level (aggregate) rather than individual patient data, the identified associations may be subject to ecological bias and should not be interpreted as evidence of individual-level relationships between tumor type or clinical characteristics and treatment effect.

Importantly, sensitivity analyses restricted to phase III trials confirmed the robustness of the primary findings, as both DFS and OS results remained consistent after exclusion of phase II studies, indicating that earlier-phase evidence did not materially influence the pooled estimates.

PD-L1 expression was evaluated heterogeneously across included trials, and the lack of standardized assays, scoring systems, and positivity thresholds limits interpretation of its potential predictive role. Although several studies reported treatment benefit across PD-L1 subgroups, the available aggregate data do not allow firm conclusions regarding whether PD-L1 expression modifies the efficacy of adjuvant ICIs.

Furthermore, we observed significant heterogeneity in treatment duration, with several trials exploring courses shorter than 1 year. However, exploratory meta-regression did not identify a significant association between treatment duration and treatment effect. Therefore, the optimal duration of adjuvant ICI therapy remains uncertain, and available aggregate data do not allow definitive conclusions regarding the impact of treatment duration on efficacy outcomes.

Treatment sequencing varied across included trials. Several studies evaluated ICI administration after surgery alone, whereas others assessed postoperative ICI following previous chemotherapy or chemoradiotherapy. This variability reflects differences in current clinical practice among tumor types and represents a potential source of heterogeneity in the pooled estimates.

Importantly, the reliance on published aggregate data rather than individual patient data introduces ecological bias, and other potential biases—such as differences in trial design, PD-L1 assessment, treatment duration—should be considered when interpreting these results.

Compared with previous meta-analyses, this study expands the available evidence by including recently published randomized trials, CTLA-4-containing regimens, exploratory dual-checkpoint blockade analyses, and longer follow-up across multiple solid tumors.

These results suggest that tumor histology and immunobiological context may contribute to variability in the observed treatment effects, although these findings remain hypothesis-generating.

The heterogeneity in results highlights significant gaps in our knowledge. Beyond trial design differences, the inconsistent success of similar agents suggests that we do not yet fully grasp the specific mechanisms driving these responses in the adjuvant setting. Future research should further investigate potential modifiers of treatment effect, including treatment duration and PD-L1 expression, using prospectively defined biomarker analyses and standardized methodologies.

Furthermore, the comparison of dual ICI therapy versus monotherapy is of great interest and warrants careful examination. While combination approaches aim to overcome resistance and enhance immune activation, they often result in a delicate balance between oncological efficacy and increased treatment-related toxicity.

An emerging challenge in the interpretation of these findings is the rapid shift toward neoadjuvant immune checkpoint inhibitor-containing regimens. The increasing adoption of neoadjuvant strategies potentially reduces the future clinical relevance of adjuvant-only approaches. This trend is not uniform across tumor types but is particularly evident in melanoma and non–small-cell lung cancer, where neoadjuvant or perioperative ICI-based approaches have demonstrated high pathological response rates and promising event-free survival signals (29–32). In urothelial carcinoma, neoadjuvant strategies are also being actively explored, although the evidence remains less mature and their integration into standard practice is still evolving (33, 34). In renal cell carcinoma and head and neck cancers, neoadjuvant immunotherapy remains largely investigational but biologically supported by activity in advanced disease and early-phase trials (35, 36). In gastric cancer, neoadjuvant immune checkpoint inhibitor–based strategies are being actively explored, with early perioperative studies in pMMR populations reporting encouraging pathological response rates (37, 38). Similarly, in hepatocellular carcinoma, early-phase neoadjuvant PD-1–based regimens have demonstrated feasibility and antitumor activity, but data are still limited (39). By administering immunotherapy before surgical resection, neoadjuvant therapy may allow for the evaluation of pathological response, and generate a more robust anti-tumor immune memory compared to postoperative administration (40–42).

Consequently, the advent of these neoadjuvant and perioperative ICI-based approaches may fundamentally alter the landscape for adjuvant trials, complicating the direct comparison of clinical outcomes. Future clinical research must account for this paradigm shift.

Finally, the future of adjuvant therapy lies depends on precision medicine, specifically the integration of circulating tumor DNA (ctDNA) as a biomarker for molecular residual disease (MRD). ctDNA has emerged as a validated prognostic and potentially predictive biomarker in several solid tumors, enabling the detection of minimal residual disease after curative-intent surgery and identifying patients at highest risk of relapse (43–45). Incorporating ctDNA monitoring into clinical protocols is essential to refine patient selection, ensuring that adjuvant immunotherapy is targeted toward those who derive the greatest benefit while sparing patients at lower risk from unnecessary toxicity.

Overall, the available data support a cautiously optimistic view of adjuvant ICIs as a clinically meaningful treatment option in multiple solid tumors. Although the benefit is not confined to a single histology and appears broadly consistent across multiple tumor types, these findings should not be interpreted as support for indiscriminate use.

Ongoing studies with standardized protocols and longer follow-up are essential to clarify long-term outcomes, refine patient selection, and better define the subgroups most likely to benefit from adjuvant immune checkpoint inhibition.

## Conclusions

Overall, the available evidence suggests a consistent but variable benefit of adjuvant immune checkpoint inhibitors across multiple solid tumors, with differences according to tumor type and clinical context. While the results support their clinical relevance, they do not justify indiscriminate use, and should be interpreted within the limits of biological heterogeneity and study-level variability. Further prospective studies with standardized designs and longer follow-up are needed to refine patient selection and optimize treatment strategies in the perioperative setting.

## Data Availability

The original contributions presented in the study are included in the article/**Supplementary Material**. Further inquiries can be directed to the corresponding author.

## Supplementary material

Supplementary Table 1 Complete search strategies for bibliographic databases and oncology conference proceedings.

Supplementary Table 2 Domain-level rationale supporting Risk of Bias 2 (ROB2) judgements of “Some concerns” for the Missing outcome data domain.

Supplementary Figure 1 Risk of bias assessment of the included randomized controlled trials.

Supplementary Figure 2 Sensitivity analyses restricted to phase III trials. Forest plots showing disease-free survival (DFS, upper panel) and overall survival (OS, lower panel).

Supplementary Figure 3 Subgroup analysis according to endpoint (DFS, upper panel; RFS lower panel).

Supplementary Figure 4 Subgroup analysis of disease-free survival (DFS) according to control group. Forest plots are shown for placebo-controlled trials (above) and observation-controlled trials (below).

Supplementary Figure 5 Sensitivity analysis of overall survival (OS) restricted to trials with median follow-up ≥24 months.

Supplementary Figure 6 Forest plot of dual-checkpoint blockade versus control arm for disease-free survival (DFS) across included trials.
