# Supplementary material for: Adjuvant immune checkpoint inhibitors across solid tumors: a meta-analysis of phase II-III randomized trials
Source: Front Oncol. 2026 Sep 7;16:1903546. doi: 10.3389/fonc.2026.1903546 (PMC13590388; doi:10.3389/fonc.2026.1903546)
Supplement: Supplementary file 1 [file Table1.docx]

**Supplementary Table 1.**

| **Database / Source** | **Search strategy** |
| --- | --- |
| PubMed | ("immunotherapy" OR "immune checkpoint inhibitor" OR pembrolizumab OR nivolumab OR atezolizumab OR durvalumab OR ipilimumab OR dostarlimab) AND ("adjuvant" OR "adjuvant therapy") AND (neoplasms OR cancer* OR tumor* OR carcinoma*) AND ("randomized controlled trial" OR randomized OR randomised) |
| Embase | (immunotherapy OR 'immune checkpoint inhibitor'/exp OR pembrolizumab OR nivolumab OR atezolizumab OR durvalumab OR ipilimumab OR dostarlimab) AND (adjuvant OR 'adjuvant therapy'/exp) AND (neoplasm* OR cancer* OR tumor* OR carcinoma*) AND (randomized OR randomised OR 'randomized controlled trial'/exp) |
| Cochrane CENTRAL | (immunotherapy OR "immune checkpoint inhibitor" OR pembrolizumab OR nivolumab OR atezolizumab OR durvalumab OR ipilimumab OR dostarlimab) AND (adjuvant OR "adjuvant therapy") AND (neoplasms OR cancer* OR tumor* OR carcinoma*) in Trials |
| ASCO Meeting Library | adjuvant AND (immunotherapy OR "immune checkpoint inhibitor" OR pembrolizumab OR nivolumab OR atezolizumab OR durvalumab OR ipilimumab) — abstracts 2015–2026 |
| ESMO Congress Abstracts | adjuvant AND (immunotherapy OR "immune checkpoint inhibitor" OR pembrolizumab OR nivolumab OR atezolizumab OR durvalumab OR ipilimumab) — abstracts 2015–2026 |
