# Supplementary material for: Adjuvant immune checkpoint inhibitors across solid tumors: a meta-analysis of phase II-III randomized trials
Source: Front Oncol. 2026 Sep 7;16:1903546. doi: 10.3389/fonc.2026.1903546 (PMC13590388; doi:10.3389/fonc.2026.1903546)
Supplement: Supplementary file 2 [file Table2.docx]

| **Study** | **Domain** | **Judgement** | **Rationale for judgement** |
| --- | --- | --- | --- |
| Eggermont et al., 2021 | Missing outcome data | Some concerns | Interim analysis of a secondary endpoint (distant metastasis-free survival); overall survival data remained immature at the data cut-off, with events still accruing and follow-up duration differing across treatment groups. |
| Kirkwood et al., 2023 | Missing outcome data | Some concerns | Pre-specified interim analysis with a short minimum follow-up (7.8 months); overall survival could not yet be evaluated and no blinded independent central review was performed, increasing the risk of incomplete or immature outcome data. |
| Motzer et al., 2023 | Missing outcome data | Some concerns | Pre-specified interim analysis (30-month median follow-up); overall survival events remained few, as acknowledged by the authors, limiting the completeness of long-term outcome data at this time point. |
| Motzer et al., 2024 | Missing outcome data | Some concerns | Third pre-specified interim analysis of overall survival (~57-month median follow-up); although more mature than earlier analyses, the outcome was still assessed at a pre-planned interim time point with ongoing accrual of events. |
| Yoon et al., 2025 | Missing outcome data | Some concerns | Post hoc subgroup analysis of KEYNOTE-716 using data from the pre-specified fourth interim analysis (not the final analysis); the authors note that recurrence event numbers were low in some tumour-location subgroups, and results should be interpreted with caution. |
| Bourhis et al., 2025 | Missing outcome data | Some concerns | First interim analysis with a wide range of individual follow-up times (9.0–66.5 months) around a 38.3-month median, indicating substantial heterogeneity in outcome data maturity across participants. |
| Chaft et al., 2026 | Missing outcome data | Some concerns | First interim analysis with a relatively short follow-up duration, explicitly noted by the authors as a factor limiting interpretation of long-term outcomes, including overall survival. |
| Goss et al., 2026 | Missing outcome data | Some concerns | The primary endpoint was evaluated only in a pre-specified subgroup (PD-L1 ≥25%, EGFR-/ALK-) rather than the full randomised population; this hierarchical design excludes part of the outcome data from the primary analysis for non-prioritised subgroups. |

***Note:*** *Only studies rated “Some concerns” on the Missing outcome data domain are listed. All remaining domains and studies were judged “Low risk”, as reported in the main Risk of Bias 2 summary table.*
